# Intralesional Injection of Mouse Mesenchymal Stem Cells Reduces IL-10 Production and Parasite Burden in *L. major* Infected BALB/c Mice

**DOI:** 10.22074/cellj.2020.6838

**Published:** 2020-09-08

**Authors:** Elham Zanganeh, Sara Soudi, Ahmad Zavaran Hosseini

**Affiliations:** Department of Immunology, Faculty of Medical Sciences, Tarbiat Modares University, Tehran, Iran

**Keywords:** *Leishmania major*, Macrophage, Mesenchymal Stem Cell

## Abstract

**Objective:**

Leishmaniasis is of public health problems, especially in endemic areas. The activation of macrophages, as the main host of leishmania and promotion of the TH1 immune responses, are the main goal of im-munotherapy methods. Recently, the immunomodulatory role of mesenchymal stem cells (MSCs) in infectious disease has been considered. Different *in vitro *studies demonstrated the immunostimulatory effect of MSCs on macrophages in response to L.major. In this study, the effect of MSCs on cutaneous leishmaniasis in BALB/c mice was assessed.

**Materials and Methods:**

To do this experimental research, BALB/c mice infected with L. major that was followed by multiple subcutaneous injections of MSCs at infection site at different intervals. Footpad thickness, spleen parasite burden, lymph node, and spleen cytokine production were measured to determine the efficacy of cell therapy.

**Results:**

Significant (P<0.05) reduction in footpad thickness and delayed wound formation was observed in MSCs treated group. The spleen of the MSCs-treated group indicated a two-fold reduction in parasite burden compared with non-treated infected mice. In addition, nitric oxide (NO), interleukin-10 (IL-10), and tumor necrosis factor-alpha (TNF-α) production of lymph node isolated cells and splenocytes changed to the benefit of macrophage activation in response to L. major in MSCs treated group. A two-fold increase in interferon-gamma (IFN-γ) production in the lymph node was determined in the MSCs-treated group.

**Conclusion:**

Although MSCs therapy could not clear the parasite, the results confirm the ability of MSCs to enhance immune responses against leishmania by induction of inflammatory responses and slowing down the spread of parasites. However, further studies needed to improve the efficacy of this method and provide a therapeutic protocol.

## Introduction

Leishmaniasis is among uncontrolled parasitic diseases, which manifests a wide variety of clinical symptoms that may lead to death. These clinical manifestations depend on Leishmania species and the host immune system (1). Cutaneous leishmaniasis is caused by Leishmania major (*L. major*) and afflicts 0.7-1.2 million cases in the world annually. Cutaneous leismaniasis has clinical manifestation from selflimiting to chronic signs in hosts, such as humans and mice (2). Due to the extracellular matrix interfaces, immediately after entering the parasite, pathogen identification, nitric oxide (NO), and hydrogen peroxide production by phagocytes is suppressed (3). In resistant mice, (C57BL/6) immune response is oriented to Th1, associated with the production of interferon-gamma (IFN-γ) and interleukin-12 (IL-12) at the site of infection, which inhibite parasite development. Susceptible mice (BALB/c) extend Th2 immune response associated with IL-4 production that terminated to systemic dissemination of the parasite (4).

Different approaches have been made for leishmaniasis treatment. The use of antibiotics, such as pentavalent antimonials, Amphotericin B, and other drugs, are among these therapeutic approaches. Because of their side effects, toxicity, cost, long term of treatment, and incomplete elimination of parasite, none of them is an ideal medicine (5). Different vaccine generations have been developed based on the killed parasite, liveattenuated parasites, and DNA vaccine; however, they failed to be used in human vaccination because of their limitations, such as lesion development or long-lasting recovery (6, 7). Immunotherapy by cytokines like IFN-γ for humans and the use of monoclonal antibodies for mice are other approaches (8, 9). Nowadays, cell therapy is a modern method to treat a broad range of infectious diseases. Some studies have shown that dendritic cells and macrophages can be used to treat leishmaniasis (7, 10).

Mesenchymal stem cells (MSCs) with self-renewal property and differentiation potential to multiple cell lineage are used for repairing tissues and rebuilding damaged organs (11, 12). They have some receptors that enable them to sense inflammatory conditions and switch inflammatory responses by secretion of soluble factors and interaction with immune cells (13, 14). In 2010, pro-inflammatory MSCs1 and antiinflammatory MSCs2 were identified, that can emerge as immune stimulator cells or immune suppressor cells, respectively, depending on cytokine milieu (15). MSCs migrate to the inflammation site and induce angiogenesis and extracellular matrix remodeling by the direction of MQ-M2 responses that terminated to repair damaged tissues (16). MSCs secrete antimicrobial peptides that enable them to fight against sepsis, acute respiratory distress syndrome, and cystic fibrosisrelated infections (17). So, MSCs are good candidates for infectious disease therapy, including Tuberculosis, Malaria, sepsis, human immunodeficiency virus (HIV), and *Trypanosoma cruzi* (18). Although the previous report rejects the effectiveness of MSCs in the treatment of leishmaniasis, our previous findings indicate that MSCs can interact with macrophages and enhance their immune response by increasing the tumor necrosis factor-alpha (TNF-α)/IL-10 ratio against *L. major* (19-21).

In the present study, the effect of the repeated local injection of MSCs was investigated on the induction of TH1/TH2 immune responses against *L. major* and parasite dissemination. For this purpose, *L. major* in their footpad injected BALB/c mice and then received adipose-derived mesenchymal stem cells (AD-MSCs) subcutaneously. Footpad swelling was monitored weekly, and *L. major* dissemination was determined by parasite burden analysis. In addition, splenocytes and lymph node cells were assessed for cytokine and NO production.

## Materials and Methods

### Animals

Six-to-eight weeks old female BALB/c mice were obtained from the Pasture Institute, Tehran, Iran. The mice were used for three purposes: *L. major* proliferation, AD-MSCs isolation and as experimental groups. The Ethics Committee of Tarbiat Modares University approved the projects with an ethical code IR.TMU.REC.1394.180.

### *L. major* culture and Leishmania antigen preparation

*L. major* promastigotes (MRHO/IR/75/ER strain) were isolated from the lymph node of the parasite reservoir BALB/c mice. Infected organs were transferred to the liquid phase of the Novy-MaccNeal-Nicolle medium. The released promastigotes were proliferated at 26°C in RPMI (Biowest, France) medium containing 5% fetal bovine serum (FBS, Gibco, USA). The stationary phase promastigotes were used to infect the mice.

In order to prepare soluble leishmania antigen (SLA), 10^9^ parasites/ml were undergone eight cycles of freezing and thawing. SLA containing supernatant was collected after centrifugation of this suspension at 8000 ɡ for 15 minutes at 4°C and stored at ˗70°C. Soluble antigen protein was measured by Bradford assay.

### Isolation of adipose-derived mesenchymal stem cells

Abdominal adipose tissues of BALB/c mice were removed aseptically. Adipose tissues were minced and digested with 0.075% type I collagenase in Dulbecco’s modified Eagle’s medium (DMEM, Gibco, USA) for 15-20 minutes at 37°C. After centrifugation at 500 g for 5 minutes, the pellets were resuspended and cultured in DMEM supplemented with 10% FBS, 2 mM L-glutamine supplementary, and 1% Pen/Strep (Gibco, USA). The cells incubated in humified air containing 5% CO_2_ at 37°C. Expanded adipose-derived MSCs (AD-MSCs) used for injection into mice and characterization.

### Characterization of adipose-derived mesenchymal stem cells

The cell surface markers of AD-MSCs at passage 3 were analyzed by Flowcytometry using monoclonal antibodies against mice CD45, CD34, CD90, CD105, CD73, and CD29 (all of them were purchased from eBioscience, USA). The cell surface markers were analyzed by the FACScalibur flowcytometer (BD Biosciences, USA) and Cyflogics software (CyFlo Ltd., Finland). The ability of AD-MSCs to differentiate into adipocyte and osteocyte was examined by Oil Red O (ORO) and Alizarin Red (AR), respectively. Adipogenic differentiation was induced by culturing AD-MSCs in a cell differentiation medium containing 10% FBS, 250 nM dexamethasone (Sigma- Aldrich, USA), 5 mM insulin (Sigma-Aldrich, USA), 0.5 nM 3-isobutyl-1 methylxanthine (Sigma-Aldrich, USA) and 100 mM indomethacin (Sigma-Aldrich, USA) for 21 days. For the purpose of osteogenic differentiation, the cells were cultured in a cell differentiation medium containing 50 mg/ml ascorbic acid-2- phosphate (Sigma- Aldrich, USA), 100 nM dexamethasone (Sigma-Aldrich, USA) and 10 mM beta-glycerophosphate (Merck, UK) for 21 days.

### Experimental groups and treatment protocol

**Fig.1:**
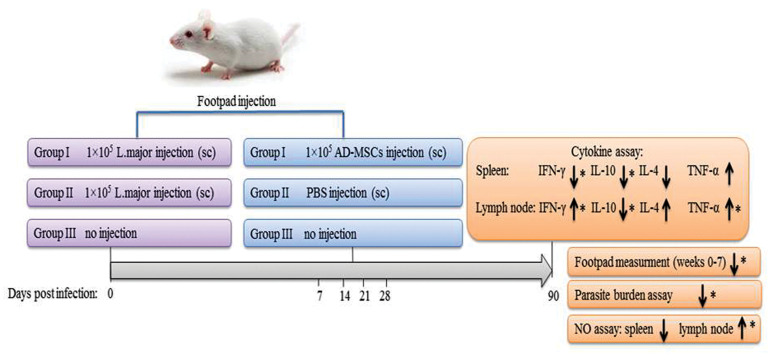
Depiction of experimental groups and treatment schedule.

The present research was based on an experimental study. Three study groups, each with 10 mice, were considered in this study. Groups I and II were infected by the footpad injection of 50 μL phosphate buffered saline (PBS, BioIdea, Iran) containing 1×10^5 ^stationary phase *L. major* promastigotes. Group III received only 50 μL of PBS by footpad injection and was kept as control. Group I was treated by the subcutaneous injection of 1×10^5 ^ of AD-MSCs at the infection site. The injection was repeated four times at 7, 14, 21, and 28 days post-infection. Group II was treated with cell-free PBS at the same periods at the infection site. The infected mice were checked daily. After observing the first symptom of swelling, footpad thickness was measured weekly by a digital caliper and noted. The experimental groups and their treatment protocol are shown in Figure 1.

### Parasite burden

The spleens of the infected mice were excised, and the number of parasites was counted by the limiting dilution assay. Briefly, 90 days post-infection, a small fragment of the infected tissues (spleen) was weighted, homogenized in 2 mL of the Schnider’s Drosophila medium (Biological Industries, USA) supplemented with 10% FBS and 0.1% Gentamicin. Serial dilution (10^-1^ to 10^-40^) was provided and transferred into 96 well culture plates in triplicate and incubated at 26°C for 2 weeks. The existence of parasite was observed by an inverted microscope. The last well containing at least a motile promastigote was noted as parasite dilution. Parasite burden was calculated with this formula:

Parasite burden=-log_10_ (parasite dilution/tissue weight)

### Nitric oxide measurement

Splenocyte and inguinal lymph node cells of five mice from each group were harvested. After red blood cell (RBC) lysis, the cells were cultured at 2×10^6 ^ cell/well of 6-well plates in the RPMI medium containing 10% FBS. Each experimental group was re-stimulated in vitro with SLA (10 μg/ml) and lipopolysaccharide (LPS, 1 μg/ml) or media. After 72 hours of the stimulation, the presence of NO was measured by the Griess method. The absorbance of the developed color was read at 540 nm and converted to NO amount (μM) according to the standard curve obtained by sodium nitrite (Merck, UK) standard concentrations. All the *in vitro* treatments were performed in triplicate.

### Cytokine detection by ELISA

Inguinal lymph node cells and splenocytes of mice from each group were isolated at 90 days post-infection. About 10^6^ cells/well were cultured in 4-well plates in triplicate and *in vitro* stimulated with either media or 10 μg/mL of SLA or 1 μg/mL of phytohemagglutinin (PHA). 72 hours after incubation, the supernatants were collected and analyzed for the presence of IL-10, IL-4, IFN-γ, and TNF-α by the ELISA method using kits from R&D Systems (Minneapolis, MN, USA) according to the manufacturer’s instructions.

### Statistical analysis

All parts of this study were repeated three times as three independent experiments. Three experimental groups, each with 10 mice, were considered in this study. So, the data are shown as mean ± standard deviation (SD) of 30 mice. Data analysis was carried out by the one-way analysis of variance (ANOVA) test according to the SPSS 13.0 (IBM, USA) software, and statistically significant differences were set at P<0.05.

## Results

### Adipose-derived mesenchymal stem cells characterization

**Fig.2:**
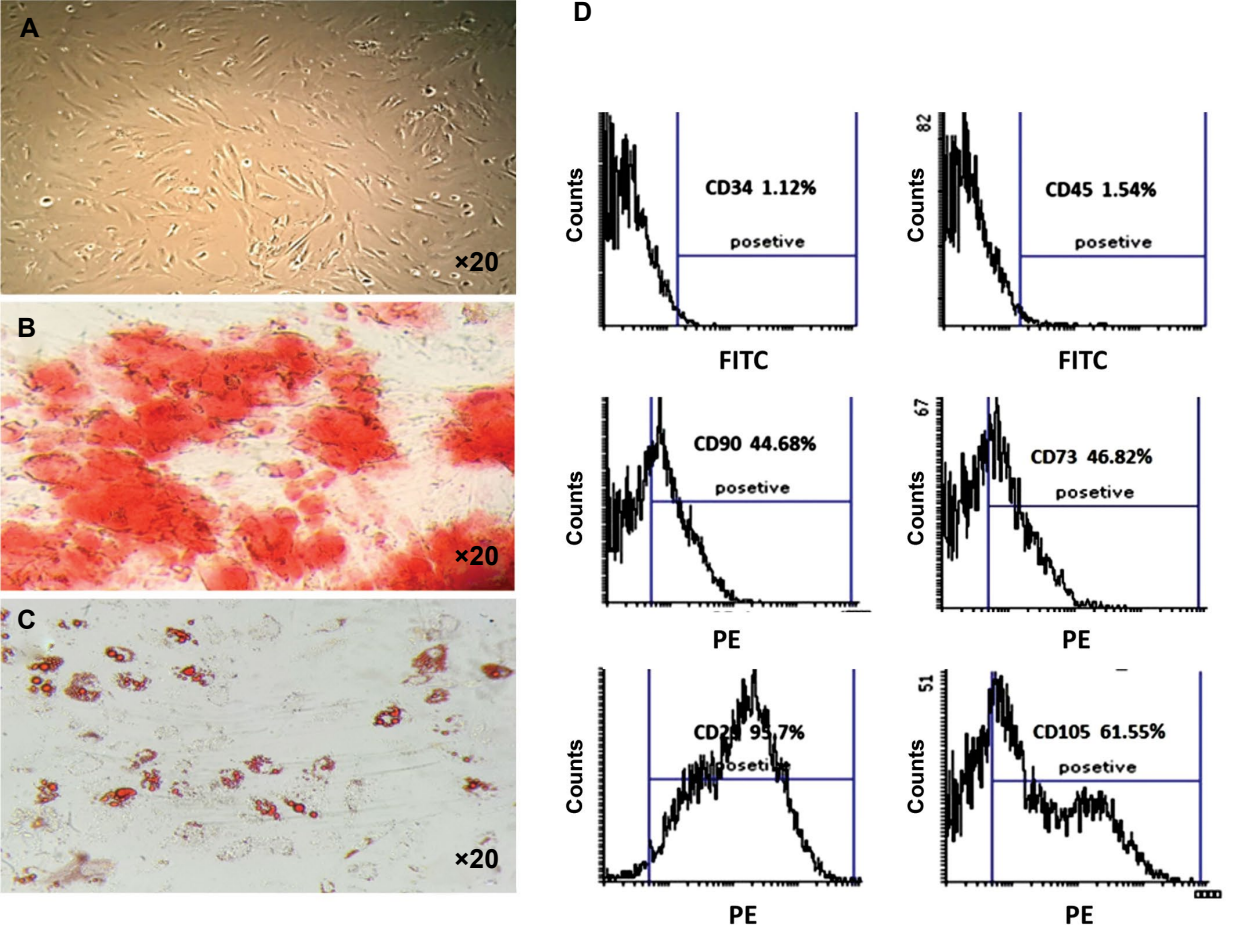
Characterization of adipose-derived mesenchymal stem cells (AD-MSCs). **A.** Fibroblast-like AD-MSCs were isolated from the BALB/c mice (×20). **B.** Alizarin red staining displayed calcium mineralization of AD-MSCs in osteocyte differentiation (×20) and **C.** Oil Red O staining display lipid droplets of AD-MSCs in adipocyte differentiation (×20). **D.** The mean percent of the cell surface markers of AD-MSCs analyzed by flow cytometry.

The AD-MSCs revealed the fibroblast-like morphology and were able to differentiate into osteocytes and adipocytes are indicated in Figure 2A, B, C, respectively. As indicated in Figure 2D, AD-MSCs expressed CD45, CD34, CD90, CD105, CD73 and CD29 cell surface markers at percentages of 1.54, 1.12, 44.68, 61.55, 46.82 and 95.7%, respectively.

### Footpad swelling measurement

**Fig.3:**
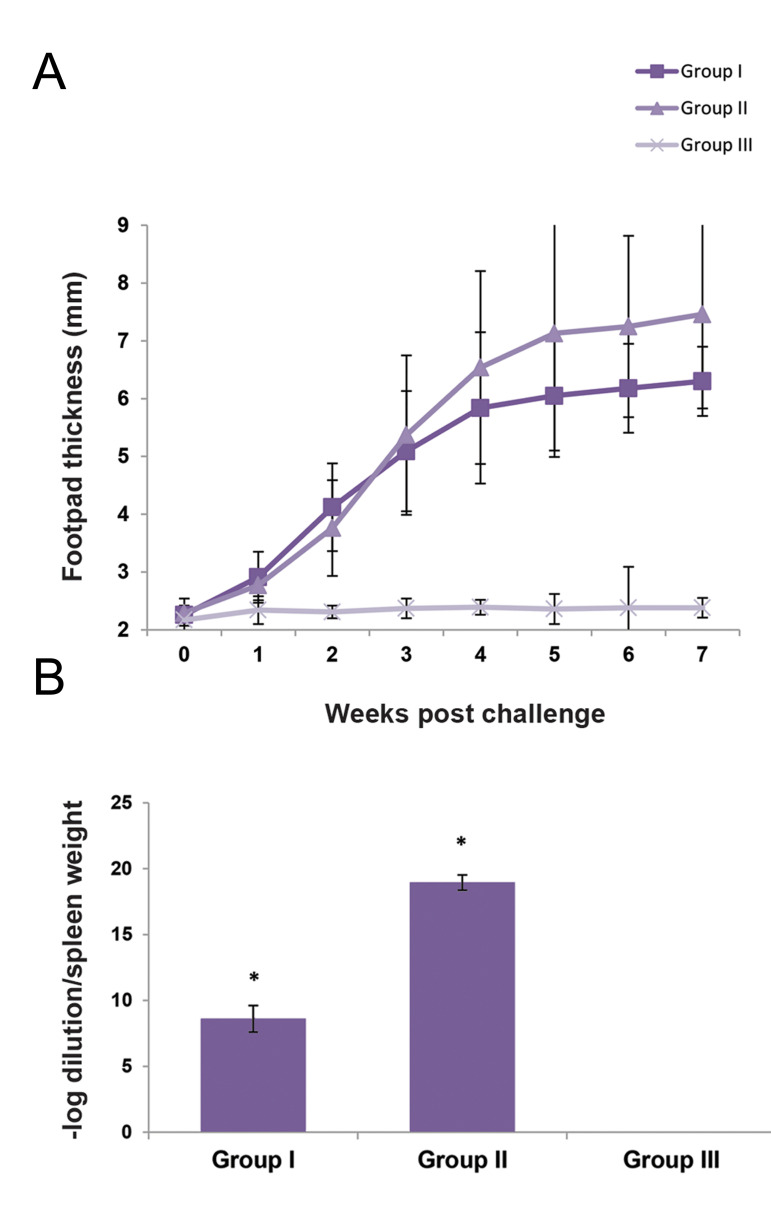
Evaluation of parasite proliferation and dissemination. **A.** Footpad thickness of the AD-MSCs treated group (group I), non-treated group (group II), and control group (group III). **B.** The number of parasites in the spleen counted by limiting dilution assay at 90 days post-infection. BALB/c mice were infected with 1×10^6^ of *L. major* parasite by footpad injection at day 0. At days 7, 14, 21, and 28 post-infection, the first infected group (group I) was treated with 5×10^5 ^ of AD-MSCs. The same volume of PBS (60 μL) was injected subcutaneously to the second infected group (group II). Data were reported as means ± SD of 30 mice. The P value was considered significantly at <0.05. A significant difference between the groups was determined by repeated measure analysis of varience (ANOVA) by the SPSS software. AD-MSCs; Adipose derived mesenchymal stem cells and PBS; Phosphate buffered saline.

The *L. major* infected mice were checked daily, and footpad thickness was measured until 7 weeks after the challenge. The first symptom of swelling without lesion was observed in the first-week post-infection in both infected groups. In the second-week post-infection, lesion development was observed in group II, while in the AD-MSCs recipient group, footpad swelling increased without lesion formation. The footpad thickness of Group II significantly (P<0.05) increased from the third week to the seventh week, as in the seventh week, lesion led to footpad necrosis. A significant decrease (P<0.05) was observed in footpad thickness between the ADMSCs treated group and non-treated group at weeks 4 to 7 post-infection. In addition, lesion formation in the AD-MSCs-treated group occurred with high latency and less severity. Group III was considered as the control without any challenge or cell treatment (Fig .3A).

### Parasite burden of the spleen

The parasite burden of the spleen was measured 90 days post-infection. The result demonstrated that the injection of AD-MSCs at the infection site affected the *L. major* proliferation. As shown in Figure 3B, parasite load in group I treated with AD-MSCs, was significantly (P<0.05) less than that of the non-treated group (group II).

### Nitric oxide production

**Fig.4:**
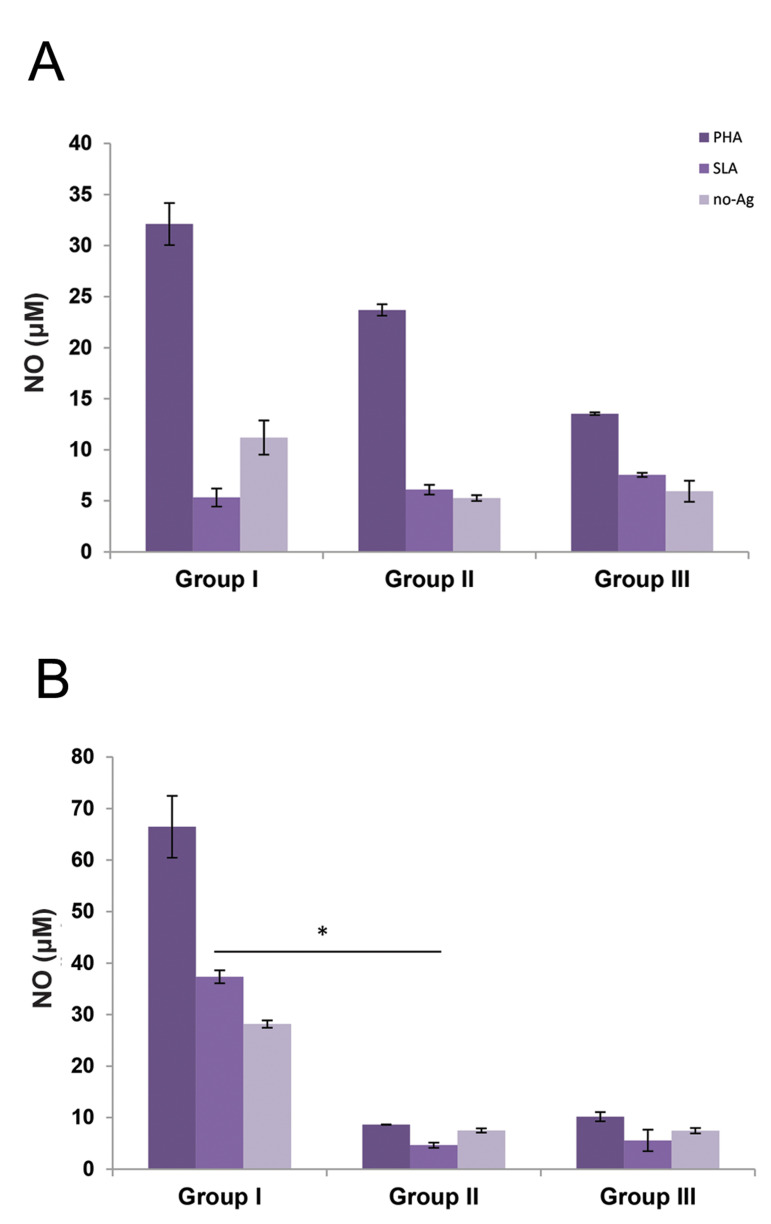
Nitric oxide (NO) production in the supernatant of experimental groups. **A.** Spleen and **B.** Lymph nodes isolated cells at 90 days post infection. Data were reported as means ± SD of 30 mice. The groups having significant differences are indicated by an asterisk (*) sign (P<0.05).

NO production was measured 90 days post-infection by the Griess method. The *in vitro* LPS treatment could induce the NO production of all the study groups compared with SLA stimulation (Fig .4A). In addition, the AD-MSCs treatment (group I) did not affect the NO production of splenocytes compared with the nontreated *L. major* infected group (group II). Unlike the spleen, the NO production of the groin lymph node isolated cells was affected by the AD-MSCs injection. NO production of both the SLA and LPS stimulated groups was significantly (P<0.05) higher in the ADMSCs treated group (P<0.05) compared with the nontreated *L. major* infected group (Fig .4B).

### Splenocyte cytokine production

**Fig.5:**
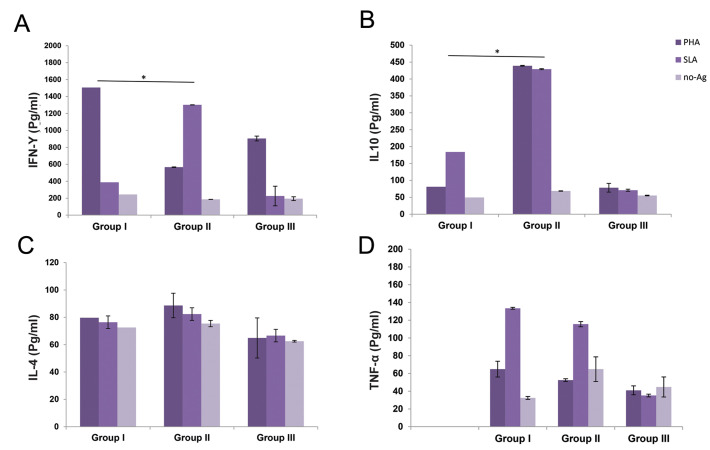
Cytokine assays in the supernatants of the splenocytes of different experimental groups. **A.** Interferon gamma (IFN-γ), **B. **Interleukin-10 (IL-10), **C.** IL-4, and **D.** Tumor necrosis factor-alpha (TNF-α). Data were reported as means ± SD of 30 mice. The groups having significant differences (P≤0.05) are indicated by an asterisk (*) sign.

Splenocyte of each group was isolated and treated with media, SLA, and PHA. After 72 hours of incubation, the supernatants were collected and evaluated for IFN-γ, IL-10, IL-4, and TNF-α cytokine analysis by the ELISA method. As indicated in Figure 5A and 5C, IFN-γ, and IL-4 production increased in the *L. major* infected groups compared with the non-infected control group. However, no significant difference was observed in terms of IL-4 production between the AD-MSCs treated (group I) and non-treated (group II) groups. IL-10 and TNF-α production increased in splenocytes of the *L. major* infected groups at 90 days post-infection. According to Figure 5B, D, the ADMSCs treatment could induce higher IL-10 and TNF-α production in response to LPS and SLA stimulation, compared with the non-treated group (group II).

### Cytokine production in the lymph node

**Fig.6:**
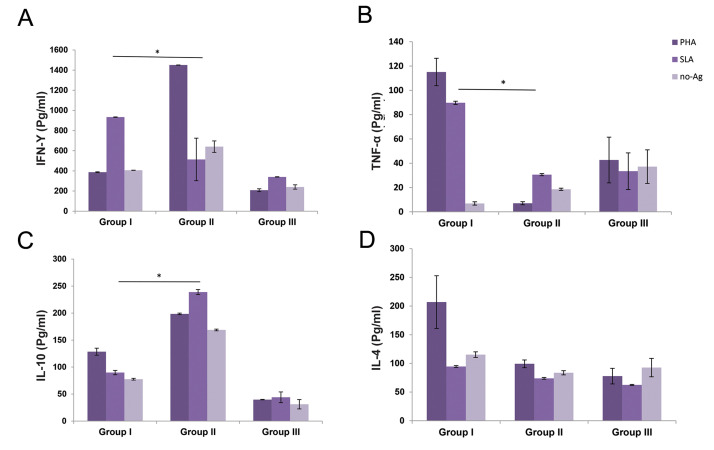
Cytokine assays in tha supernatants of the lymph node isolated cells at 90 days post-infection. **A. **Interferon gamma (IFN-γ), **B.** Interleukin-10 (IL-10), **C.** IL-4, and **D.** Tumor necrosis factor alpha (TNF-α). Data were reported as means ± SD of 30 mice. The groups having significant differences (P.0.05) are indicated by an asterisk (*) sign.

The lymph node cells of each group were isolated and then treated with media, SLA, or PHA. After 72 hours of incubation, IFN-γ, IL-10, IL-4, and TNF-α cytokine production was measured by the ELISA method. As indicated in Figure 6A and 6B, the production of IFN-γ and TNF-α was induced significantly (P<0.05) in the lymph node of the AD-MSCs-treated group compared with the non-treated infected mice. Although interleukin-10 production was more in the *L. major* infected group than in control, the increase in the ADMSCs treated group (group I) was significantly lower than that in the non-treated group (group II) (Fig .6C). Moreover, there was no significant difference in IL-4 production of both the AD-MSCs treated and nontreated *L. major* infected groups (Fig .6D).

## Discussion

Leishmaniasis is one of the neglected parasitic diseases that is transmitted by sandflies in tropical areas (22). These intracellular pathogens spread by targeting immune cells and induction of inappropriate immune responses that terminated to loss of proper functioning of the infected organs (23). Drug toxicity and the emergence of resistance, along with the lack of approved protective vaccines, indicated the need for new therapeutic approaches (7, 24). The ability of MSCs to modulate immune responses made them as therapeutic tools of inflammatory disorders, including infectious disease (25). Different studies demonstrated the direct or indirect effect of MSCs therapy on T-cell count and differentiation in infectious disease. Allam et al. (26) showed that MSCs therapy of HIV patients induces a significant increase in naive and memory CD4+T cells and restores their ability to produce IL-2 and IFN-γ in response to HIV antigens. In another study, Thakur et al. (27) introduced the protective role of MSCs in malaria infection by the suppression of IL10 and regulatory T-cell differentiation and induction of IL-12 production. In the current study, the potential effects of the adipose-derived MSC therapy evaluated on parasite dissemination and induction of TH1/TH2 responses in a murine model of cutaneous leishmaniasis. AD-MSCs considered in this research because the preparation of human adipose tissue is easy, non-invasive, and ethically accepted (28). In addition, adipose-derived MSCs have a more homogeneous population than those derived from bone marrow (29).

Following the footpad injection of leishmania, the immune cells migrated into the infection site, and inflammatory responses appeared, resulting in an increase in the thickness of the footpad. If the protective innate immune responses developed, the proliferation of the parasite would be limited, and the inflammatory responses would decrease. In other words, if the unprotected inflammatory responses were created, the accelerated proliferation of the parasite, ulcers, and necrosis would appear at the infection site (30). The current results demonstrated the efficacy of AD-MSCs administration for the control of lesion formation and footpad necrosis. In addition, the footpad thickness in the AD-MSCs treated group was significantly less than that the non-treated group on weeks 4 to 7 post-infection, indicating a decrease in the severity of inflammation. The lower parasite load in the spleen of mice that received AD-MSCs demonstrated the control of *L. major* proliferation at the infected site and its dessemination to other organs.

Activation of NO synthase and respiratory burst terminated to produce NO and reactive oxygen species (ROS) is among the important mechanisms that macrophages use to kill Leishmania parasites (31). Different studies indicated that the amount of NO production by macrophages and neutrophils was associated with resistance to leishmaniasis and control of parasite proliferation (32). The obtained results represented the 

enhanced production of NO by the lymph node isolated cells in the AD-MSCs treated group. This increase can be one of the reasons for the lower parasite burden, and slow wound formation in the footpad of the AD-MSCs treated group compared with the non-treated one. IL-10 cytokine production is one of the important indicators of failure or victory against leishmaniasis. Different studies demonstrated that an increase in IL-10 production affected the anti-leishmania activity of innate immune cells and inhibited Th1 cell development and IFN-γ production (33, 34). In addition, the persistence of IL-10 at the infected skin causes the persistence of *L. major* after clinical cure and reactivation of the disease (35). In the current study, the significant reduction in IL-10 production in both the spleen and lymph node of the AD-MSCs treated group demonstrated the efficacy of MSCs in the treatment of leishmaniasis. TNF-α is another protective cytokine, the importance of which differs depending on parasite strains (36). However, the ability of TNF-α in the induction of NO and the increased risk of animal death in the absence of TNF-α reflected its protective role (37). The ELISA study showed that the level of TNF-α production in the spleen of the infected mice was higher than that of the lymph node. The local administration of AD-MSCs can significantly increase the amount of this cytokine in the lymph node. All the experiences in the study of leishmaniasis in resistant and susceptible mice indicated a key role in inducing Th1 immune responses in controlling and treating leishmaniasis (38). In this research, the ADMSCs treatment could induce IFN-γ production of the lymph node at the infected site through attenuation of IL-10 production. Considering that in the previous study, MSCs therapy could not have a positive role in the control of leishmaniasis, different results of the current study indicate the importance of the injection route and the frequency of injection in the success of cell therapy. Since the present study does not investigate the mechanism of MSCs’ action in reducing IL-10, it is not possible to discuss it clearly. However, it seems that multiple injections of MSCs help to generate sequential waves of pro-inflammatory type I MSCs (15) that terminated to induction of TNF-α, reduction in IL-10, and control of parasite dissemination. Different studies have shown the efficacy of multiple MSCs injection in the improvement of the disease.

## Conclusion

Multiple intralesional injections of MSCs can induce the formation of protective responses at the early phase of *L. major* infection. However, this positive effect was not long-lasting and ultimately led to parasite dissemination and death of susceptible infected mice. We need to find a solution that can help to maintain the effectiveness of stem cells and remove barriers in this way.
